# A Community-wide Media Campaign to Promote Walking in a Missouri Town

**Published:** 2005-09-15

**Authors:** Ricardo J Wray, Keri Jupka, Cathy Ludwig-Bell

**Affiliations:** Saint Louis University School of Public Health; Health Communication Research Laboratory, Saint Louis University School of Public Health, St Louis, Mo; Southern Illinois University Edwardsville, Edwardsville, Ill

## Abstract

**Introduction:**

Engaging in moderate physical activity for 30 minutes five or more times per week substantially reduces the risk of coronary heart disease, stroke, colon cancer, diabetes, high blood pressure, and obesity, and walking is an easy and accessible way to achieve this goal. A theory-based mass media campaign promoted walking and local community-sponsored wellness initiatives through four types of media (billboard, newspaper, radio, and poster advertisements) in St Joseph, Mo, over 5 months during the summer of 2003.

**Methods:**

The *Walk Missouri* campaign was conducted in four phases: 1) formative research, 2) program design and pretesting, 3) implementation, and 4) impact assessment. Using a postcampaign-only, cross-sectional design, a telephone survey (N = 297) was conducted in St Joseph to assess campaign impact. Study outcomes were pro-walking beliefs and behaviors.

**Results:**

One in three survey respondents reported seeing or hearing campaign messages on one or more types of media. Reported exposure to the campaign was significantly associated with two of four pro-walking belief scales (social and pleasure benefits) and with one of three community-sponsored activities (participation in a community-sponsored walk) controlling for demographic, health status, and environmental factors. Exposure was also significantly associated with one of three general walking behaviors (number of days per week walking) when controlling for age and health status but not when beliefs were introduced into the model, consistent with an a priori theoretical mechanism: the mediating effect of pro-walking beliefs on the exposure–walking association.

**Conclusion:**

These results suggest that a media campaign can enhance the success of community-based efforts to promote pro-walking beliefs and behaviors.

## Introduction

Sedentary lifestyles contribute to chronic diseases, such as cardiovascular disease, cancer, and diabetes, and to risk factors including obesity (1). In 2003, the majority of U.S. adults (52.8%) were not physically active at levels that promote health (2). In Missouri, 54.9% of the adult population in 2003 failed to get enough physical activity to provide any health benefits (2). *Healthy People 2010* includes several goals that seek to increase physical activity levels in the United States (3).

Walking is an easy and accessible way to achieve the recommended amount of daily activity (4,5). Thirty minutes of brisk walking five or more times per week substantially reduces the risk of developing or dying of coronary heart disease, stroke, colon cancer, diabetes, high blood pressure, and obesity (6).

Known determinants of physical activity participation fall into multiple categories, including demographics (7), psychosocial factors (8-10), social support (7), and neighborhood and other environmental factors (11-13). Media-based interventions have been implemented to promote physical activity in recent years, but three recent reviews of media-based programs have disagreed on their potential to change behavior (14-16). Our review of studies on eight media campaigns in five countries offers moderate evidence of impact on behavior change. The data suggest success in reaching audiences, with exposure rates (percentage of a population that reports seeing or hearing campaign messages) ranging from 38% to 90% (17-22) and campaign-message recall rates (the percentage of the population that is able to recall a specific campaign message) ranging from 23% (19) to 30% (17). These studies also report shifts in cognition related to moderate physical activity. For example, in a study in Australia, 62% of individuals exposed to media campaigns reported awareness of the benefits of moderate physical exercise (compared with 29% of control populations) (21). In a national study in New Zealand, the percentage of adults intending to be more active increased from 2% in 1999 to 9% in 2002, following a media campaign (22).

The data on behavior change, however, are mixed. Campaigns in the United Kingdom (18) and Scotland (23) resulted in no increase or a negligible increase in physical activity despite moderate levels of exposure to media campaigns. In Brazil, a citywide campaign achieved a campaign-message recall rate of 56% and was associated with a reduction in sedentary lifestyles (19). Two national campaigns in Australia in 1990 and 1991 were shown to increase intention to engage in physical activity and physical activity behavior in older adults in the first year, but physical activity levels reached a plateau in the second year (24). A national campaign in New Zealand was associated with a 5% increase in the proportion of walkers in a national survey, but the gain declined to baseline in the second year (22). A campaign in New South Wales, Australia, was shown to increase knowledge about the benefits of physical activity and lead to increases in self-efficacy for physical activity; residents were twice as likely to engage in physical activity as residents in other states (20). A 6% national decline in physical activity improved in New South Wales to 4.4% during the period of the campaign (21). Finally, in Wheeling, WVa, a campaign reaching 90% of the population affected stages of change as well as perceived behavioral control and intention. In this quasi-experimental study, the proportion of walkers in the intervention community increased after the campaign more than it increased in a comparison community where no campaign was implemented (25).

Some consensus for an integrated approach to increase physical activity — including environmental and policy changes, community-based programs, and media campaigns — has emerged (14-16,26). For example, in its recent evaluation of interventions promoting physical activity, the Centers for Disease Control and Prevention's (CDC's) *Guide to Community Preventive Services* *(Community Guide)* strongly recommends community-wide initiatives that include informational components, but it finds insufficient evidence to recommend media-only approaches (16). It is not clear which, if any, informational elements should be included in an integrated campaign to promote physical activity. This gap in knowledge has led to research designed to address two important questions. Can mass media-based interventions support community-based activities? If so, how do media campaigns contribute to community-based interventions designed to promote physical activity? To answer these questions, we describe the design, development, implementation, and impact assessment of *Walk Missouri,* a mass media campaign designed to promote walking in a Missouri town.

## Methods

### Campaign design and development

The *Walk Missouri* campaign was conducted in four phases: 1) formative research, 2) program design and pretesting, 3) implementation, and 4) impact assessment. Elements of the health belief model (HBM) (27) provided a framework for the effort from formative research through assessment. Perceived susceptibility and perceived severity, two elements of the HBM, were not included in this study because their predictive ability has been questioned in other research (28,29); perceived barriers and perceived benefits were included in the framework because they have been shown to be strongly correlated with physical activity behavior (7).

#### Phase 1: Formative research

We conducted 24 focus groups in 2001 in both midsize and large metropolitan areas across Missouri. The demographic characteristics of the focus group members participating in the formative research are shown in Table 1. Focus group questions were designed to identify perceived benefits of and barriers to walking as well as possible cues to action. Focus group findings indicated that participants responded more readily to messages emphasizing ways to overcome barriers and the short-term benefits of walking rather than long-term health benefits. These findings informed messages that emphasized the short-term positive outcomes of walking and identified strategies to overcome obstacles.

#### Phase 2: Message development and pretesting

Behavioral messages for the media campaign were developed as cues to action, reminding Missourians of the short-term health benefits of walking (e.g., losing weight), the social benefits (e.g., spending time with loved ones), and the pleasure benefits (e.g., having fun). Messages also communicated ways to overcome barriers (e.g., providing ideas on how to incorporate walking into a busy schedule). Messages included phrases and themes drawn from the formative research focus groups. Messages used a question-and-answer format, with a message answering one of four questions: when, where, why, or with whom do you walk? Answers were emphasized more than questions (i.e., were spoken first in radio advertisements or were set in larger typeface on billboards and posters and in newspaper advertisements). Messages also used the phrase "do it" to pique curiosity.

Print, radio, and television messages were developed and tested with 16 focus groups representing a range of audience segments across Missouri. Table 1 shows the characteristics of this second group of participants. The campaign strategy was received positively by the focus groups, and message materials were selected for the campaign.

#### Phase 3: Implementation

The multimedia campaign took place during a 5-month period from May through September 2003. The media plan consisted of billboard, newspaper, radio, and poster advertisements. (Television spots were not used because of the expense of buying airtime.) The strategy for media placement was to achieve the greatest visibility at the outset, in May and June, followed by reduced numbers of advertisements from July through September. In a press conference to kick off the campaign, local political leaders and coalition partners announced the *Walk Missouri* campaign to local radio, television, and newspaper outlets. Table 2 shows the amount and cost of media space and time purchased. Table 3 presents a complete list of messages included in all types of media in the St Joseph, Mo, campaign.

The campaign was designed to reach adult residents of St Joseph, Mo. A midsize town with a population of 84,909 in 2003 (30), St Joseph is located about 45 miles north of Kansas City, Mo. The community was chosen because of its cooperation during the first two stages of the study and its commitment to physical activity initiatives. St Joseph had already developed local initiatives to increase physical activity, including an extensive network of walking trails and an active worksite wellness coalition *(Get Movin' St. Joe)* led by the Buchanan County Health Department, the YMCA, and Heartland Health, the owner of a local hospital. The *Get Movin' St. Joe* coalition was active in the community, working with worksites, schools, and athletic organizations for at least 1 year before the *Walk Missouri* campaign. Although the worksite wellness coalition advertised its events through its affiliated groups, it had not engaged in mass media advertising. Consistent with the *Community Guide* recommendation for community-wide campaigns (16), the *Walk Missouri* campaign tapped into these local initiatives, augmenting available resources and increasing campaign reach.

Take a tour of selected print materials from the *Walk Missouri* campaign

**Figure:**
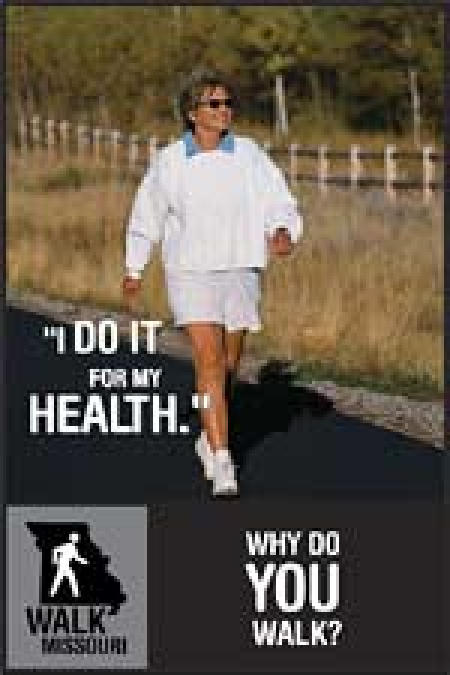
*Walk Missouri* poster (PDF 146K) VISUAL: A smiling woman walking down a countryside road. HEADLINE COPY: “I do it for my health. Why do you walk?” VISUAL: WALK MISSOURI (LOGO)

**Figure:**
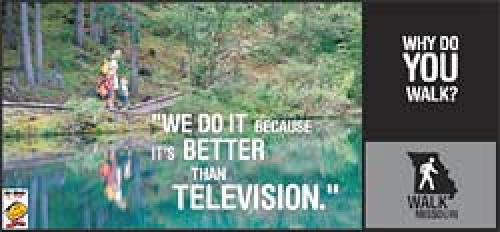
*Walk Missouri* billboard advertisement (PDF 676K) VISUAL: A mother and child walking together through a forest, beside a lake. HEADLINE COPY: "We do it because it's better than television. Why do you walk?" VISUAL: WALK MISSOURI (LOGO)

**Figure:**
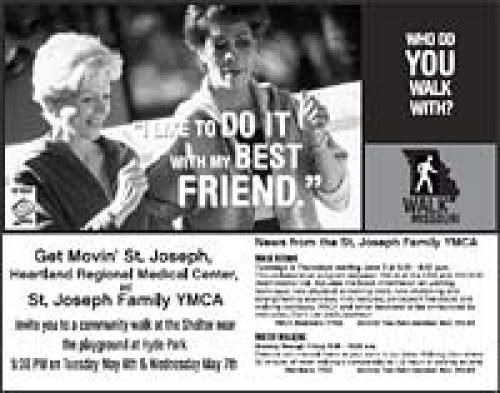
*Walk Missouri* newspaper advertisement (PDF 2.5Mb) VISUAL: Two women walking briskly together. HEADLINE COPY: "I like to do it with my best friend. Who do you walk with?" COPY:
Get Movin' St. Joseph, Heartland Regional Medical Center, and St. Joseph Family YMCA Invite you to a community walk at the shelter near the playground at Hyde Park 5:30 PM on Tuesday May 6th & Wednesday May 7th**News from the St. Joseph Family YMCA****Walk Reebok** Tuesdays & Thursdays starting June 3 at 5:30 – 6:30 p.m. This collaborative program between YMCA of the USA and REEBOK International Ltd. includes the latest information on walking technique, new physical screening tests, new stretching and strengthening exercises, mini lectures, participant handouts and walking workouts. YMCA and other locations to be announced by instructor. Don't just walk, workout! YMCA Members: Free Activity Fee (Non-member fee): $48.00**Water Walking** Monday through Friday 9:30 – 10:30 a.m. Exercise with minimal stress to your joints in our Water Walking class where 30 minutes of water walking is comparable to 1 1/2 hours of walking on land. Members: Free Activity Fee (Non-member fee): $48.00 VISUAL: WALK MISSOURI (LOGO) Get Movin' St. Joe (LOGO)

The *Get Movin' St. Joe* coalition organizers participated in implementing the *Walk Missouri* campaign. Local walking resources and activities organized by *Get Movin' St. Joe* were incorporated into *Walk Missouri* newspaper and radio advertisements. For example, the *Get Movin' St. Joe* logo was incorporated into the *Walk Missouri* campaign advertisements. Local collaborators increased visibility of the *Walk Missouri* campaign by distributing and displaying *Walk Missouri* campaign posters in community centers, businesses associated with the worksite wellness program, and other locations across the town. In this way, the *Walk Missouri* media effort helped to advertise community-sponsored walking activities and resources while capitalizing on local efforts to expand *Walk Missouri* campaign reach.

#### Phase 4: Impact assessment

The objectives of the *Walk Missouri* campaign were as follows:

- To increase knowledge and positive beliefs about the social and short-term health benefits as well as pleasures of walking;
- To increase knowledge and positive beliefs about ways to overcome barriers to walking;
- To increase participation in community walking and wellness activities; and
- To increase amount and frequency of walking.

The figure shows the conceptual framework of the evaluation, including the hypothesis that exposure to the *Walk Missouri* campaign could achieve a direct effect on walking behaviors, indicated by the arrow linking exposure to behaviors. Alternately, there might be an indirect effect of exposure on behaviors, mediated by pro-walking beliefs, indicated by the arrows linking exposure with beliefs and beliefs with behaviors. In testing for either of these effects, we controlled for likely moderating factors.

**Figure:**
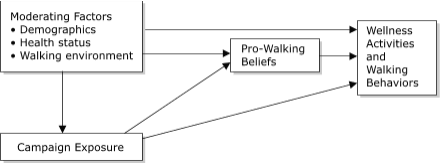
Conceptual framework for *Walk Missouri* media campaign evaluation, St Joseph, Mo, 2003.

### Evaluation methods

#### Survey design and sample

The Saint Louis University Institutional Review Board approved this study. A postcampaign-only design was used: phone numbers for residents living within the city of St Joseph were purchased from a market research firm, and a random-digit–dial telephone survey was conducted. Individuals were eligible to participate if they identified themselves as adult (aged 18 years or older) residents of St Joseph. Trained callers conducted the interviews between July 31 and October 31, 2003. The survey required an average of 15 minutes to complete. Individual numbers were dialed numerous times before being eliminated from the survey. A total of 297 interviews were completed with the funds available for evaluation.

#### Measures

*Exposure.* As in the evaluation of other media campaigns (31), various campaign exposure measures were used to evaluate the *Walk Missouri* campaign, including campaign-exposure questions, media-type–exposure questions, and dose-exposure questions. Both prompted and unprompted questions were asked. (The Appendix provides all survey items used in the analysis.) To discern media-type dose exposure, individuals were first asked if they had been exposed to any campaign advertisements through billboards, radio, or newspapers or if they had seen any campaign posters or news stories about the campaign. (News stories were initiated by local newspapers in response to the press conference and the campaign.) Individuals who answered in the affirmative for a media type were then asked how many times they had been exposed to that type. For example, respondents who answered *yes* for billboards were asked in how many locations they had noticed billboards sponsored by the campaign (with answers ranging from one to six billboards). Respondents who answered *yes* for radio were asked how many times they had heard radio advertisements sponsored by the campaign (with survey items offering ranges of 1 to 5, 6 to 10, 11 to 20, 21 to 50, 51 to 100, or more than 100 times).

Two variables were developed for analysis of exposure: a four-level dose-exposure scale and a dichotomous variable (exposed and unexposed). The four-level dose-exposure scale summed the five media-type dose-exposure items in the survey. A higher value on this scale signifies either more types of media through which the campaign was seen or heard or a greater number of messages seen or heard through fewer types of media. Because the scale was highly skewed toward no exposure, the scale was recoded as a four-category variable (none, low, medium, and high exposure) to make coefficients more stable. A value of 1 (low) signifies that the respondent reported seeing one billboard, newspaper advertisement, or newspaper story; heard only five or fewer radio advertisements; or saw only five or fewer posters. A value of 2 (medium) signifies that the respondent reported exposure to the campaign through two or three types of media or exposure to more advertisements through one or two types of media. A value of 3 (high) signifies that the respondent reported exposure on four or more types of media or exposure to more advertisements on fewer types of media. Because of varying ranges within survey items and different kinds of exposure to messages on different types of media, it was not practicable to convert the scale into levels of frequency of exposure. The four-level scale was used to assess associations of amount of exposure with study outcomes; it was also recoded into a new dichotomous variable (exposed and unexposed) to test for group differences.

*Beliefs. *The survey asked participants 12 questions about their opinions of exercise using a 5-point Likert scale. Four subscales were computed from nine survey items to measure theoretical belief constructs. Despite a small number of items in each subscale (only two or three), the Cronbach a calculated for each subscale was near or higher than minimum desired level of a = .70 for social benefits (α = .66), pleasure benefits (α = .58), health benefits (α = .73), and social support (α = .60). (A fifth subscale for overcoming barriers was dropped from the analysis because of an unacceptably low Cronbach α of .46.) Subscales were computed using belief items that were recoded to three levels because the individual items and the resulting scales had normal distributions and would provide more stable results in statistical analyses. Strongly disagree, disagree, and neutral were consolidated as one value coded as 1; agree was coded as 2; and strongly agree was coded as 3. To make them comparable, each subscale was computed to three value scales by dividing the summed scale by the original number of items. To test for a mediating effect of beliefs on the association between exposure and behaviors, a single all-beliefs scale was computed from all 12 belief items in the survey (Cronbach α = .75).

*Behavior measures.* The survey asked six walking-behavior questions. Three dichotomous (yes or no) measures inquired about walking and wellness activities sponsored by *Get Movin' St. Joe*. One dichotomous (yes or no) and two continuous measures of walking behavior were adapted from physical activity measures in the 2000 Behavioral Risk Factor Surveillance System (BRFSS).

*Moderators.* Various measures were included to control for possible alternative explanations for evaluation results. Demographic measures included sex, age, race, and level of education. Health-related measures included health status, medical diagnosis of chronic disease or overweight, medical advice to walk more, and recent injury. Perceived safety of the participant's walking environment was assessed using six Likert items. Cronbach α for the scale was .63.

*Analysis.* Our conceptual framework (Figure) offers the hypothesis that exposed individuals are more likely to hold beliefs consistent with campaign themes and more likely to engage in walking activities than individuals not exposed to the campaign and controlling for likely alternative explanations. In the multivariate analyses, we controlled for moderators that were significantly associated with outcomes. In addition, we hypothesized that beliefs had a mediating effect on the association between exposure and behaviors.

Group differences for the exposed and unexposed portions of the sample were assessed using two-tailed *t* tests for ordinal and continuous outcomes and chi-square tests for dichotomous outcomes. Associations between amount of exposure and outcomes were assessed using the Spearman rank correlation (ρ). For multivariate analyses, linear regression was used for ordinal and continuous outcomes, and logistic regression was used for dichotomous outcomes. When beliefs were significantly associated with behaviors, stepwise regression was used to test a mediating effect of beliefs on any associations between exposure and behaviors.

## Results

### Response rate

During data collection, 4668 phone numbers were used, 2866 of which were out of scope (e.g., businesses, out-of-service numbers, numbers failing to be answered after multiple calls). Of the remaining 1802 numbers, 1461 refused participation before we were able to determine eligibility. Of the remaining 341, five respondents were aged younger than 18 years, bringing our total number of eligible respondents to 336. The total number of completed interviews was 297, resulting in a cooperation rate of 88% (297/336). Compared with the Council of American Survey Research Organization (CASRO) response rates of 54.6% found for the BRFSS Missouri, which include estimates of eligible households among households for which eligibility was not determined, our response rate was low at 17% (32). However, our response rate proved to be better than the rate of 9.1% provided for random-digit–dial surveys tracked by the Market Research Association (33). Low response rates for random-digit–dial surveys are increasingly a problem for evaluators of health promotion interventions (34). 

### Survey findings

#### Sample characteristics and exposure levels

The sample had more women and was older, better educated, and more diverse than the U.S. census indicates for this area (Table 4). Thirty-two percent of the sample reported exposure to the campaign through news and advertising media. Exposure levels by type of media ranged from 7% (newspaper advertisements) to 13% (newspaper articles and billboards). Among respondents reporting exposure to the campaign through different types of media, the median number of advertisements reported for each type of media was one to five posters, two newspaper advertisements, two newspaper stories, one billboard, and six to 10 radio advertisements. The exposed respondents (32%) were distributed evenly to low (11%), medium (10%), and high (11%) levels within the dose-exposure scale. A separate analysis found no demographic differences between exposed and unexposed groups, nor was any association found between dose exposure and demographic characteristics.

#### Beliefs

On a scale of 1 to 3, with 2 indicating agree, survey participants rated all beliefs as approximately 2 (Table 5). The mean for the all-beliefs scale was 4, also equivalent to agree, on the 5-point scale. The exposed group reported greater agreement with two of the four belief subscales (social benefits and pleasure benefits) than the unexposed group at a statistically significant level. Amount of exposure was associated with three of four subscales (social, pleasure, and health benefits) and the all-beliefs scale at a statistically significant level.

#### Behaviors

The exposed group reported a greater level of participation in three of six wellness or walking behaviors than the unexposed group at a statistically significant level. Amount of exposure was associated with the same three behaviors at a statistically significant level. Two of the outcomes were wellness behaviors: participation in a community-sponsored walk or participation in a health fair. The third outcome was a general walking behavior: the number of days per week the respondent walked at least 10 minutes.

#### Association of exposures, beliefs, and behaviors

Beliefs and behaviors associated with campaign exposure at the bivariate level were selected for multivariate analysis, controlling for variables associated with the dependent variable in bivariate analyses. Campaign-dose exposure remained associated with two of the four belief subscales (social benefits and pleasure benefits) when controlling for likely confounding factors (Table 6). The association of campaign-dose exposure with the health benefits subscale and all-beliefs subscale was not statistically significant when controlling for other factors.

Campaign-dose exposure remained associated with participation in a community-sponsored walk at a statistically significant level when controlling for educational level. In this analysis, the odds ratio of dose exposure was 2.14 (confidence interval, 1.04–4.41; *P* = .04); exposed respondents were more than twice as likely to participate in the community-sponsored walks than unexposed respondents. In another multivariate analysis (not shown), dose exposure was not associated with participation in community-sponsored health fairs when controlling for other factors.

Campaign-dose exposure was associated with the number of days per week walking at a statistically significant level when controlling for age and health status (Table 7). However, when the all-beliefs scale was introduced in the second step of the linear regression, the coefficient for campaign exposure lost statistical significance.

## Discussion

Impact assessment of media campaigns seeks to answer the question "Did exposure to the campaign lead to changes in beliefs and behavioral outcomes?" The evidence presented here shows that exposure to the *Walk Missouri* campaign had limited effects, producing small increases in positive walking beliefs and behaviors among residents of St Joseph. Effect sizes were small, with Spearman ρs of between 0.13 and 0.18 for statistically significant associations of beliefs and behaviors with campaign-dose exposure. Among exposed respondents, 4.3% reported participation in community-sponsored walks, compared with 0.5% of unexposed respondents. Exposed respondents reported walking for at least 10 minutes per day 5.2 days of the week, compared with 4.5 days per week for unexposed respondents.

When an external control community and baseline measures are not possible for assessing the impact of media campaigns, alternative approaches can offer evidence of effects when they show 1) moderate levels of campaign reach; 2) associations of exposure with behaviors, controlling for alternative explanations; 3) and confirmation of an a priori hypothesis positing mediation of exposure–behavior associations by beliefs promoted by the campaign (35). The *Walk Missouri* evaluation set out to provide such evidence. First, survey respondents reported a moderate level of exposure to the campaign, with about one in three respondents reporting some exposure. This level of exposure was near the 36% average for health-behavior media campaigns found in a recent meta-analysis (36). Second, exposure was significantly associated with three of six walking-behavior measures, and one association remained when controlling for several known predictors: demographic characteristics, health status, perceptions of the walking environment, and health beliefs.

Third, in addition to differences between exposed and unexposed groups, there was a dose-response relationship between exposure and outcomes, with higher levels of agreement on beliefs and positive walking behavior corresponding to higher levels of exposure.

Fourth, the association of exposure and number of days walking was mediated by health beliefs, providing evidence of a theoretically informed causal mechanism. For one wellness behavior — participation in community-sponsored walking activities — beliefs did not mediate the association with exposure. We conclude that campaign information on walking opportunities increased knowledge about these activities, leading to a slight increase in walking.

The single-site, postcampaign-only design limited the power of the study in several ways. Lack of an external control community and baseline measures may have weakened the study's internal validity. We cannot rule out the possibility of reverse causal direction — that walking adherents were more likely to pay attention to and recall the campaign. Nor can we conclude that the associations we found were not the result of other unmeasured third factors. The low response rate for our random-digit–dial survey may have introduced selectivity bias into the sample and limited our ability to generalize even to the medium-size midwestern town of St Joseph. Self-report measures are vulnerable to socially desirable responses, although there is no evidence that this necessarily contributed to differences between groups.

Acknowledging these limitations and caveats, our study provides information for public health communication researchers and practitioners about the potential for media interventions to promote physical activity. The study elucidates how a media campaign can contribute to a community-sponsored effort to promote walking behavior.

### Evidence despite a simple design

Elements of this single-site, postcampaign-only, cross-sectional design support our claim of limited effect. Careful and multiple measurements of exposure allowed for the creation of an unexposed comparison group. Two measures of exposure confirmed both group differences and dose-response associations of exposure and outcomes. A dose-response relationship suggested a possible causal relationship between exposure to the media campaign and increased likelihood of undertaking walking behaviors (37). We account for likely alternative explanations for associations of exposure with outcomes by including moderating factors in multivariate analysis. Empirical support for a theoretical mechanism is established in a test of the mediating effect of pro-walking beliefs on the association of campaign exposure and walking behavior. Combined, these results strengthen our claim of limited effect by ruling out alternative explanations and supporting an a priori theoretical approach that underlies the campaign strategy and study design (35).

### Support for the message strategy

The association of exposure with social and pleasure benefits suggests that the campaign was most successful in communicating these ideas. Although health benefits, social support, and overcoming barriers were also included in the messages, they did not appear to have as much of an impact on the intended audience.

### Successful integration into local activities

Originally envisioned and designed as a stand-alone media campaign, *Walk Missouri* was successfully integrated into local community-sponsored activities, consistent with recommendations from the literature (14-16,26), including the *Community Guide,* which strongly recommends programs that include informational promotional components. The local community coalition welcomed the opportunity to serve as the pilot community for the campaign, indicating that campaign themes and messages complemented their activities. It was not difficult to integrate information about local community activities and the coalition into *Walk Missouri* message materials, including scheduled walks and other wellness activities, lists of participating sponsors, and the *Get Movin' St. Joe* logo. Adaptation of the *Walk Missouri* campaign materials provided practical, useful information grounded in local events and organizations and proved pertinent for residents of St Joseph.

The scale of the initiative was closer to the community-based initiative of Wheeling, WVa (25), than the national and statewide campaigns implemented in New Zealand and Australia (20,22), all of which included investments in community-based programs. The *Walk Missouri* campaign incorporated information about community-sponsored activities, but *Walk Missouri* did not otherwise fund or support local activities. Nonetheless, it can be argued that in actively linking with community-based initiatives, the campaign fit more closely within the category of community-wide activities than in the media-alone category suggested by the *Community Guide* (16).

### Media contributions to behavior change

The limited effect achieved by the *Walk Missouri* campaign is similar to the results of other media campaigns promoting general health behaviors as well as the few media campaigns that have promoted walking. The difference in wellness behaviors between exposed and unexposed groups was small (between 3 and 10 percentage points), consistent with research on the effects of health campaigns on behavior (36), and with previous efforts to promote physical activity through media interventions (20,22).

The study provides tentative evidence of independent and complementary effects of media campaigns on community-based interventions. We argue that the *Walk Missouri* media campaign expanded the reach of the local initiative, *Get Movin' St. Joe.* Originating as a worksite-wellness effort and a community coalition, *Get Movin' St. Joe* benefited from the visibility provided by the many posters, newspaper advertisements, billboards, and radio spots of *Walk Missouri*, and as a result, *Get Movin' St. Joe* reached a wider audience.

The study design does not allow us to differentiate between effects of media exposure and exposure to other community-sponsored activities; however, the evidence does permit us to discern how the campaign affected walking behavior. The evidence suggests that the campaign achieved behavioral results in two ways. First, campaign exposure was associated with participation in community-sponsored walks, consistent with a direct effect of exposure on behavior. Integration of information about community-sponsored walking activities and resources into media messages rendered them practical and useful. In this way, the media campaign was *complementary* to the community-sponsored events and may well have boosted attendance.

Second, the association of exposure with number of days of walking was mediated by pro-walking beliefs, consistent with an indirect effect of exposure on behavior. This finding suggests an independent effect of the campaign on walking behavior. General walking behavior did not rely on coalition activities. More importantly, the pro-walking beliefs that mediated the exposure–behavior association were consistent with campaign themes highlighted in *Walk Missouri*. These results begin to distinguish the mechanisms by which communication elements of a community-wide campaign contribute to increases in physical activity directly by advertising events and indirectly through changed beliefs and attitudes about walking.

### Important impact on the margin

For a limited time, residents of St Joseph who were exposed to the campaign may have walked almost 1 day more per week than residents who were not exposed. More people may have attended coalition events because they heard about them through radio spots. Incremental increases in levels of physical activity at the population level contribute to major gains in public health (38).

By accounting for alternative explanations and substantiating a theory-based mechanism for impact, a simple study design with a small sample can provide persuasive evidence of campaign effects. The results of this small study with limited resources provide encouraging evidence that a media campaign can enhance the success of community-based efforts to promote positive walking beliefs and behaviors.

## Figures and Tables

**Table 1:** Demographic Characteristics of Focus Group Participants, *Walk Missouri* Campaign, 2001–2002

**Characteristics**	**Formative Research Focus Groups** **(N = 24)**	**Pretest Focus Groups** **(N = 16)**
No. participants	174	118
Female, %	Not collected	80
Age, median, y (range)	44 (18-83)	46 (18-83)
Education, mean, y (range)	14.3 (8-16+)	14.5 (9-16+)
Household income, mean, $	30,000-39,999	30,000-39,999

**Race and ethnicity %**		

White	83	83
African American	15	14
Native American	1	1
Hispanic	1	1
Other	0	1

**Table 2:** Media Purchases for *Walk Missouri* Campaign, St Joseph, Mo, 2003

**Type**	**Amount Spent, $**	**No. Advertisements**
Billboards	2760	8
Newspapers	5862	16
Radio	9876	1296
Posters	800	200
**Total**	**19,298**	**1520**

**Table 3:** Messages Promoted in *Walk Missouri* Campaign, St Joseph, Mo, 2003

**Type**	**Messages Used**
Billboards	I like to do it with my best friend. *Who do you walk with?* Sunday afternoons are a family affair. *When do you walk?* We like to do it in nature's backyard. *Where do you walk?* We do it because it's better than television. *Why do you walk?* It's like recess for grown-ups. *Why do you walk?*
Radio	We do it as a church group. I do it with my co-workers on my lunch hour. We do it together. I do it with friends at the gym. *Who do you walk with?* I do it first thing in the morning to start the day off right. I do it on my lunch hour; it feels great. I do it on my way to work and on my way home. I do it after dinner to help me wind down. *When do you walk?* I do it around my neighborhood every morning. I do it at the mall. I do it at the gym. I do it at the park. I do it around the softball field while my daughter practices. *Where do you walk?* We do it because a family who plays together, stays together. I do it because it's easy to fit into my busy schedule. I do it to feel better and have more energy. I do it to lose weight. I do it because it’s fun. *Why do you walk?*
Newspaper	My co-worker and I keep each other on track. *Who do you walk with?* Sunday afternoons are a family affair. *When do you walk?* We like to do it in nature's backyard. *Where do you walk?* I do it to set a good example. *Why do you walk?*
Posters	It's great for catching up with my buddy. *Who do you walk with?* We like to do it on cool, cloudy days. *When do you walk?* We like to do it in nature. *Where do you walk?* I do it for my health. *Why do you walk?*

**Table 4:** Demographic Characteristics of Respondents and Results of Telephone Survey to Assess *Walk Missouri* Media Campaign Impact on Community, St Joseph, Mo, 2003

**Characteristic (No. Respondents)**	**Measure**
Female (295)	62%
Age, mean, y (290)	47±17.43

**Education (290)**	

Some or completed high school	41%
Some college or received college degree	47%
Some graduate school or completed graduate degree	11%
Not sure	1%

**Race and ethnicity (296)**	

African American	4%
White	84%
Hispanic	3%
Native American	3%
Asian or other	6%

**Levels of exposure to media in general**	

No. hours per week listening to radio, median (296)	2
No. days per week reading newspaper, median (296)	7

**Frequency of observing types of media^a^**	

Posters (296), median	Sometimes
Newspaper ads (293), median	Often
Billboards (296), median	Sometimes

**Exposure to *Walk Missouri* Campaign**	**Measure**

Exposed through media (297)	32%

**Posters**	

Respondents exposed	11%
Median no. seen	1-5

**Newspaper ads**	

Respondents exposed	7%
Median no. seen	2

**Newspaper stories**	

Respondents exposed	13%
Median no. seen	2

**Radio ads**	

Respondents exposed	8%
Median no. heard	6-10

**Billboards**	

Respondents exposed	13%
Median no. seen	1

**Dose-exposure scale (297)^b^**	

0 (none)	68%
1 (low)	11%
2 (medium)	10%
3 (high)	11%

a 1–4 scale where 1 = never, 2 = rarely, 3 = sometimes, 4 = often.

b Dose-exposure scale is the recoded sum of the items measuring number of advertisements to which respondents reported exposure; a higher value signifies either a greater number of types of media through which the campaign was seen or heard or a greater number of messages seen or heard through fewer types of media.

**Table 5:** Telephone Survey Results by Level of Exposure to *Walk Missouri* Media Campaign, St Joseph, Mo, 2003

	**Grand Mean Score** ** (SD)**	**Unexposed** **Respondents ** ** (Mean Score)**	**Exposed** **Respondents** ** (Mean Score)**	**Test of Difference^a^ ** ** (*P* value)**	**Low Exposure** ** (Mean Score)**	**Medium Exposure** ** (Mean Score)**	**High Exposure** ** (Mean Score)**	**Test of Association ** ** (*P* value)**

**Belief subscale^b^ (no. respondents)**								

Social benefits (293)	1.95 (0.55)	1.90	2.05	t_291_ = −2.24 (.03)	1.83	2.16	2.18	ρ = 0.15 (.01)
Pleasure benefits (295)	2.25 (0.46)	2.20	2.36	t_293_= −2.84 (.005)	2.31	2.28	2.48	ρ = 0.18 (.002)
Health benefits (290)	2.22 (0.51)	2.18	2.30	t_288_ = −1.81 (.07)	2.28	2.25	2.35	ρ = 0.12 (.04)
Social support (290)	1.67 (0.58)	1.64	1.72	t_288_ = −1.04 (.30)	1.72	1.71	1.73	ρ = 0.03 (.56)
All beliefs (279)	3.82 (0.49)	3.78	3.89	t_277_ = −1.76 (.08)	3.86	3.89	3.92	ρ = 0.13 (.04)

**Behaviors (no. respondents)**								

Participated in sponsored walk (*Walk Missouri* or *Get Movin' St. Joe*) (295)	2%	0.5%	4.3%	χ^2^_1_ = 5.4 (.02)	3%	3%	6%	ρ = 0.14 (.01)
Participated in worksite wellness activities (295)	11%	10%	13%	χ^2^_1_= 0.6 (.44)	13%	0%	24%	ρ = 0.06 (.21)
Participated in health fair sponsored by a local health care provider (295)	13%	10%	20%	χ^2^_1_ = 5.9 (.02)	28%	7%	24%	ρ = 0.13 (.02)
Walked for at least 10 min at a time during usual week (296)	89%	89%	88%	χ^2^_1 _= 0.01 (.94)	84%	90%	90%	ρ = 0.01 (.93)
No. days per week walked at least 10 min at a time (260)	4.73	4.52	5.2	t_7 _= −2.34 (.02)	5.19	5.12	5.27	ρ = 0.16 (.01)
On days walked at least 10 min, total minutes walked on a scale of 1-6^c^ (248)	3	3.54	3.63	t_5_ = −0.38 (.71)	3.48	3.96	3.48	ρ = 0.02 (.74)

a All t tests are unpaired, two-tailed tests.

b Belief subscales were computed by summing belief items that were recoded to three levels (with strongly disagree, disagree, and neutral consolidated as 1, agree as 2, and strongly agree as 3). The subscales were then computed to three value scales by dividing the summed scale by the original number of items. The all-beliefs scale was computed by summing 12 original 5-point Likert items, then dividing by 12.

c Survey provided six categories (Appendix) with three representing 30–39 minutes.

**Table 6:** Linear Regression Analysis for Belief Subscales, *Walk Missouri* Campaign, St Joseph, Mo, 2003

**Independent Variables**	**Social Benefits^a^**			**Pleasure Benefits^b^**			**Health Benefits^c^**			**All Beliefs^d^**		
	**B (95% CI)^e^**	**SE**	***P***	**B (95% CI)**	**SE**	***P***	**B (95% CI)**	**SE**	***P***	**B (95% CI)**	**SE**	***P***
Age	−0.005 (−0.01 to 0.003)	.004	.20	Excluded^f^			Excluded			Excluded		
School	Excluded			0.06 (0.003 to 0.12)	.03	.04	0.08 (−0.02 to 0.18)	.05	.11	Excluded		
Sex	Excluded			Excluded			0.51 (0.16 to 0.87)	.18	.01	1.46 (0.15 to 2.77)	.67	.03
Health status	0.11 (−0.02 to 0.25)	.07	.10	0.14 (0.04 to 0.25)	.05	.01	0.22 (0.04 to 0.40)	.09	.02	1.26 (0.61 to 1.92)	.33	<.001
Told overweight	−0.28 (−0.59 to 0.03)	.16	.08	Excluded			Excluded			Excluded		
Advised by doctor to walk	0.02 (−0.29 to 0.32)	.15	.90	Excluded			Excluded			Excluded		
Injury	Excluded			Excluded			Excluded			−2.58 (−4.68 to −0.49)	1.07	.02
Walking environment	0.05 (0.01 to 0.08)	.02	.01	0.05 (0.02 to 0.08)	.01	.001	0.08 (0.03 to 0.12)	.02	.001	0.44 (0.27 to 0.61)	.09	<.001
Campaign dose exposure	0.16 (0.05 to 0.28)	.06	.01	0.14 (0.04 to 0.24)	.05	.006	0.11 (−0.05 to 0.28)	.08	.17	0.44 (−0.16 to 1.05)	.31	.15

a Belief in social benefits = 2.73 ? (.005 × Age) + (.11 × Health status) ? (.28 × Told overweight) + (.02 × Advised by doctor) + (.05 × Walking environment) + (.16 × Campaign dose exposure). Adjusted R^2^ = 0.09.

b Belief in pleasure benefits = 2.67 + (.06 × School) + (.14 × Health status) + (.05 × Walking environment) + (.14 × Campaign dose exposure). Adjusted R^2^ = 0.11.

c Belief in health benefits = 3.59 + (.08 × School) + (.51 × Sex) + (.22 × Health status) + (.08 × Walking environment) + (.11 × Campaign dose exposure). Adjusted R^2^ = 0.09.

d All walking beliefs = 30.88 + (1.46 × Sex) + (1.26 × Health status) ? (2.58 × Injury) + (.44 × Walking environment) + (.44 × Campaign dose exposure). Adjusted R^2^ = 0.18.

e B indicates unstandardized regression coefficient; CI, confidence interval.

f Variables were included in analyses only when they were associated with the dependent variable at the bivariate level.

**Table 7:** Stepwise Linear Regression for Number of Days Walked per Week, *Walk Missouri* Campaign, St Joseph, Mo, 2003^a^

**Independent Variables**	**First Step**			**Second Step**		

	**B (95% CI)^b^**	**SE**	***P***	**B (95% CI)**	**SE**	***P***
Age	−0.02 (−0.04 to −0.003)	0.01	.02	−0.02 (−0.04 to −0.006)	0.01	.01
Health status	0.62 (0.35 to 0.89)	0.14	<.001	0.51 (0.24 to 0.79)	0.14	<.001
Campaign dose exposure	0.29 (0.04 to 0.53)	0.12	.02	0.24 (−0.01 to 0.48)	0.12	.054
All beliefs scale	Excluded in first step			0.08 (0.04 to 0.3)		<.001

a Number of days walked per week = ?.133 ? (.02 × Age) ? (.52 × Health status) + (.24 × Campaign dose exposure) + (.08 × All-beliefs scale). Adjusted explained variance (R^2^) = 0.15.

b B indicates unstandardized regression coefficient; CI, confidence interval.

## Appendix: Survey Questions for the *Walk Missouri* Media Campaign

### Exposure questions

1a. In the past 30 days, do you remember seeing any posters sponsored by the *Walk Missouri* campaign displayed in the St Joe area?

1. Yes
2. No (skip to next question)
3. Not sure (skip to next question)
4. Refuse (skip to next question)

1b. In the last 30 days, in how many different locations have you noticed a poster sponsored by the *Walk Missouri* campaign in your community? (*Read the ranges if respondent has trouble answering.*)

1. 1 to 5
2. 6 to 10
3. 11 to 20
4. 21 to 50
5. 51 to 100
6. Over 100

2a. In the last 30 days, do you remember seeing any newspaper advertisements sponsored by the *Walk Missouri* campaign?

1. Yes
2. No (skip to next question)
3. Not sure (skip to next question)
4. Refuse (skip to next question)

2b. In the last 30 days, how many times have you seen *Walk Missouri* advertisements in your local newspapers?

1. 1 time
2. 2 times
3. 3 times
4. 4 times
5. 5 or more times (specify)
6. Not sure

3a. In the last 30 days, do you remember seeing any local news stories about *Walk Missouri* or *Get Movin’ St. Joe*?

1. Yes
2. No (skip to next question)
3. Not sure (skip to next question)
4. Refuse (skip to next question)

3b. How many news articles have your seen or read in the last 30 days that mention *Walk Missouri* or *Get Movin’ St. Joe*?

1. 1 time
2. 2 times
3. 3 times
4. 4 times
5. 5 or more times (specify)

4a. In the last 30 days, do you remember hearing radio ads sponsored by *Walk Missouri*?

1. Yes
2. No (skip to next question)
3. Not sure (skip to next question)
4. Refuse (skip to next question)

4b. In the last 30 days, how many times have you heard radio ads sponsored by *Walk Missouri*?

1. 1 to 5 times
2. 6 to 10 times
3. 11 to 20 times
4. 21 to 50 times
5. 51 to 100 times
6. Over 100 times

5a. In the last 30 days, do you remember seeing any billboard advertisements sponsored by *Walk Missouri*?

1. Yes
2. No (skip to next question)
3. Not sure (skip to next question)
4. Refuse (skip to next question)

5b. In the last 30 days, in how many different locations have you noticed a billboard sponsored by the *Walk Missouri* campaign in your community?

1. 1
2. 2
3. 3
4. 4
5. 5
6. 6 or more times (specify)

### Belief questions

I would now like to ask you a few questions about your opinions of exercise.

Please indicate whether you

1 = Strongly agree
2 = Agree
3 = Neither agree nor disagree
4 = Disagree
5 = Strongly disagree
6 = Not sure
7 = Refuse

1. Exercise is a good way to spend time with people who are important to me.
2. Exercise is a good time for me to catch up with my friends.
3. People who are important to me encourage me to walk regularly.
4. Walking is good way to ensure I have good health.
5. Walking is good way to control my weight.
6. I can walk almost anywhere at any time.
7. In order to get the benefits of walking, it has to be hard work.
8. Walking helps me deal with stress.
9. Walking is a good way to enjoy the outdoors.
10. It is important to me that my family and friends know I walk for exercise.
11. I can find time to walk.
12. Walking can be fun.

### Behavior questions

1. In the past 30 days, have you participated in a walk sponsored by *Walk Missouri* or *Get Movin’ St. Joe*?
  1. Yes
  2. No
2. In the past 30 days, have you participated in any wellness activities at your worksite?
  1. Yes
  2. No
3. In the past 30 days, have you attended a health fair sponsored by a local health care provider such as the hospital or health department?
  1. Yes
  2. No
4. In a usual week, do you walk for at least 10 minutes at a time while at work, for recreation, for exercise, to get to and from places, or for any other reason?
  1. Yes
  2. No
5. In the past week, how many days per week did you walk for at least 10 minutes at a time?
  0 = 0 days
  1 = 1 day
  2 = 2 days
  3 = 3 days
  4 = 4 days
  5 = 5 days
  6 = 6 days
  7 = 7 days
6. In the past week, how many days per week did you walk for at least 10 minutes at a time?
  1 = 10–19 minutes
  2 = 20–29 minutes
  3 = 30–39 minutes
  4 = 40–49 minutes
  5 = 50–59 minutes
  6 = 60 minutes or more

### Moderator questions: demographics, health status, and walking environment

1. What year were you born? 19__
2. What is the highest year of school completed?
  1. Some high school
  2. High school diploma or equivalent
  3. Some college
  4. Associate's degree
  5. Bachelors degree
  6. Some graduate work
  7. Graduate or advanced degree
  8. Not sure/silent code
3. Would you characterize yourself as . . . ?
  1. African American
  2. Asian
  3. Caucasian
  4. Hispanic
  5. Native American
  6. Other (specify)
  7. Not sure/silent code
4. I am sorry, but I have to ask all of the questions. Are you . . .
  1. Male
  2. Female
5. Would you say that in general your health is …
  1. Excellent
  2. Very good
  3. Good
  4. Fair
  5. Poor
  6. Don’t know
6. Have your ever been told by your doctor that you are overweight or have a chronic disease such as heart disease, high blood pressure, high cholesterol, diabetes, arthritis, osteoporosis, or another chronic disease?
  1 = Yes
  0 = No
  2 = Not sure/don’t know
7. Have you ever been told by your doctor that you should walk or exercise more?
  1 = Yes
  0 = No
  2 = Not sure/don’t know
8. There are many types of injuries, such as a broken bone, a cut, a bruise, a pulled muscle, or a sprained ankle. In the last 30 days, have you been injured badly enough that you needed to change your daily activities for at least 1 week?
  1 = Yes
  0 = No
  2 = Not sure/don’t know
9. Please indicate whether you . . .
  1 = Strongly agree
  2 = Agree
  3 = Neither agree nor disagree
  4 = Disagree
  5 = Strongly disagree
  6 = Not sure/silent code
  - There are many places to walk (for example, a store, a workplace, etc.) within easy walking distance from my home.
  - I often walk to places near my home.
  - There are good sidewalks on most of the streets in my community.
  - There are interesting things to look at while walking in my neighborhood.
  - I feel safe walking around my neighborhood.
10. Do you find your neighborhood . . .
  1 = Very pleasant
  2 = Somewhat pleasant
  3 = Not very pleasant
  4 = Not at all pleasant
  5 = Not sure/silent code
