# Supplementary material for: A Community-wide Media Campaign to Promote Walking in a Missouri Town
Source: Prev Chronic Dis. 2005 Sep 15;2(4):A04. (PMC1432093)
Supplement: Supplementary file 1 [file 05_0010_01.pdf]

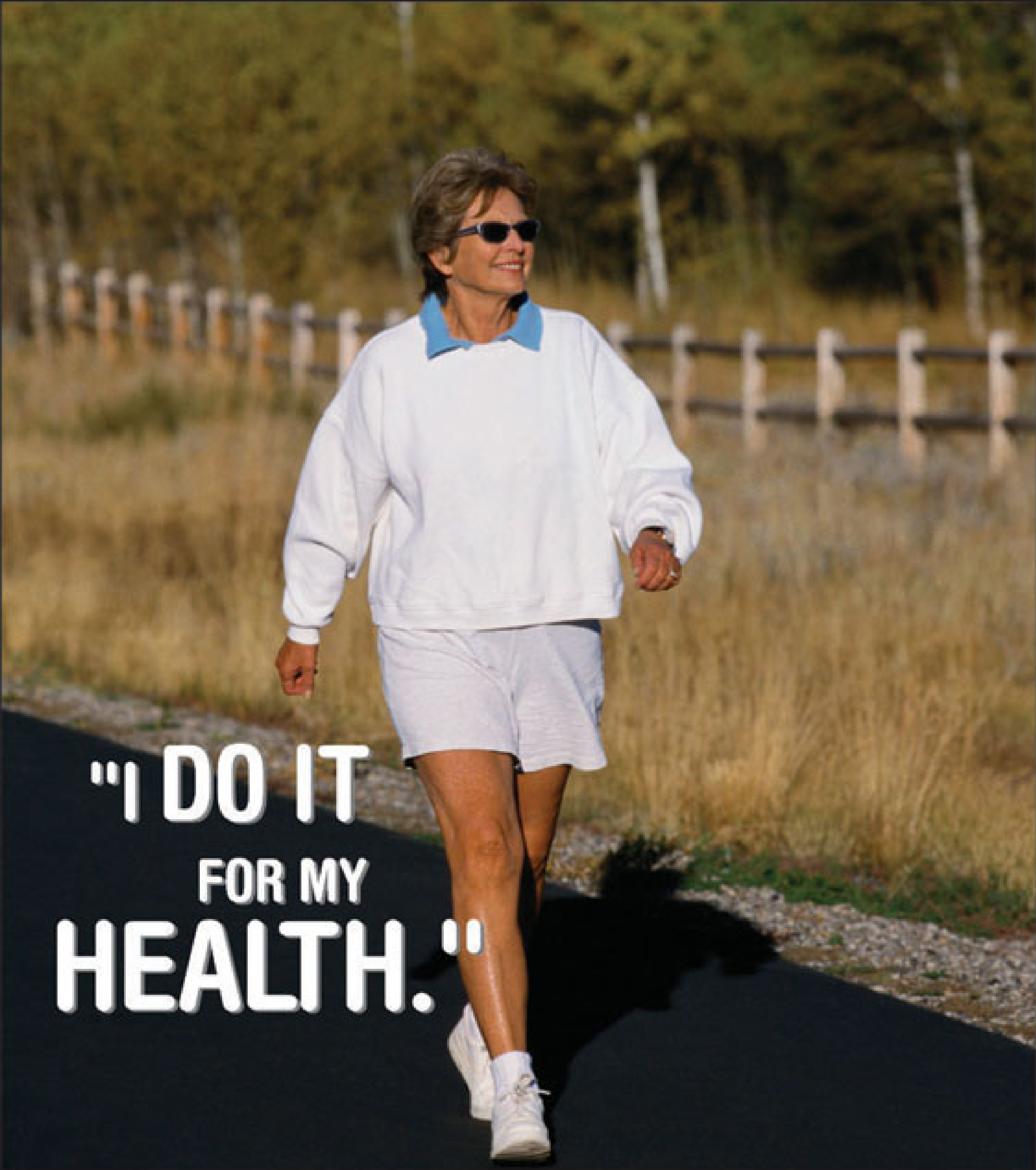

WALK
MISSOURI

WHY DO
YOU
WALK?
