# Supplementary figures and images for: A Community-wide Media Campaign to Promote Walking in a Missouri Town
Source: Prev Chronic Dis. 2005 Sep 15;2(4):A04. (PMC1432093)

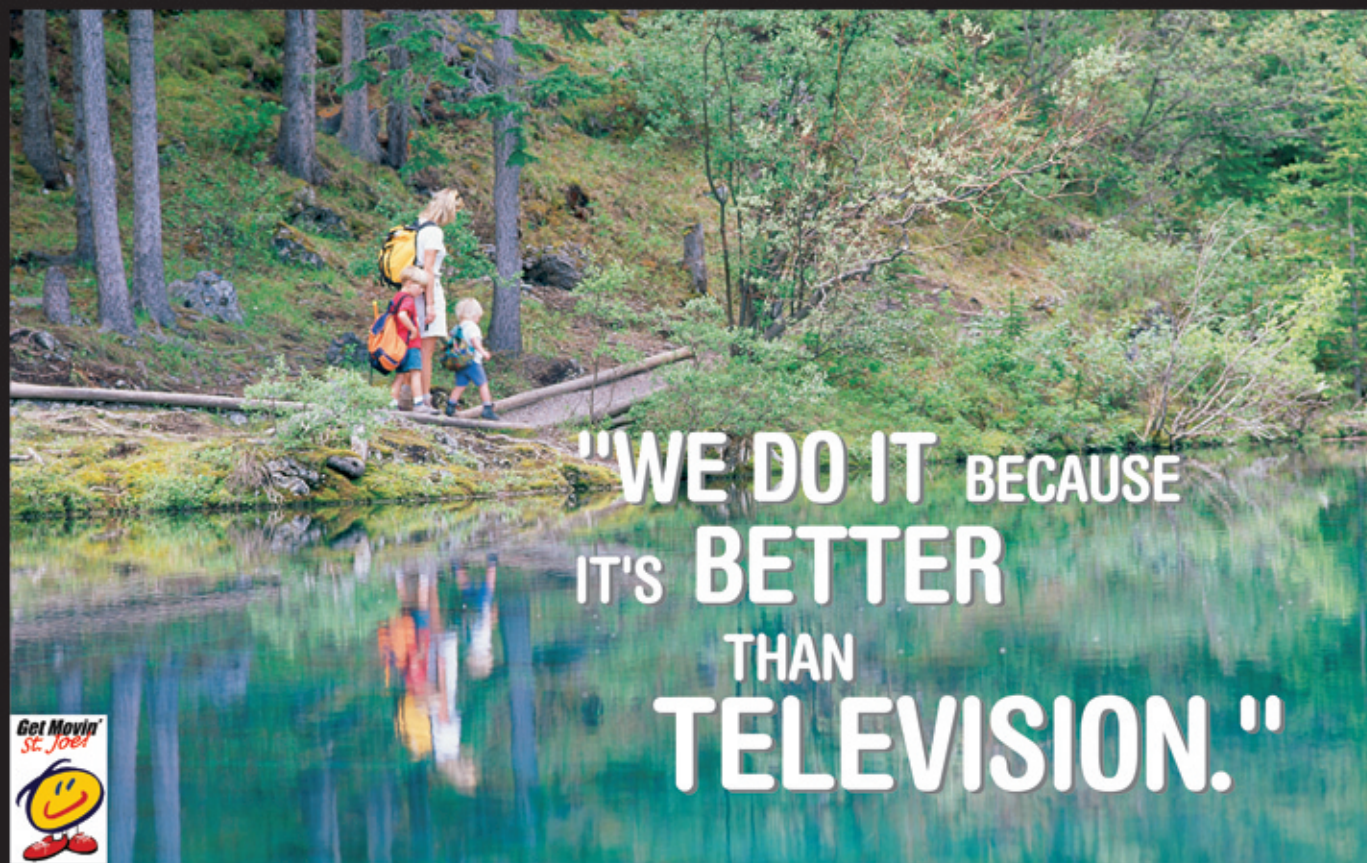

"WE DO IT BECAUSE  
IT'S BETTER  
THAN  
TELEVISION."

WHY DO  
**YOU**  
WALK?

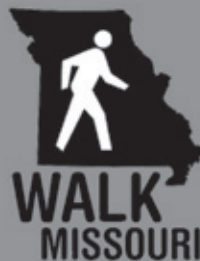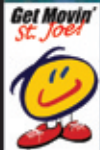

Supplement: Supplementary file 2 [file 05_0010_02.pdf]
