# Supplementary material for: A Community-wide Media Campaign to Promote Walking in a Missouri Town
Source: Prev Chronic Dis. 2005 Sep 15;2(4):A04. (PMC1432093)
Supplement: Supplementary file 3 [file 05_0010_03.pdf]

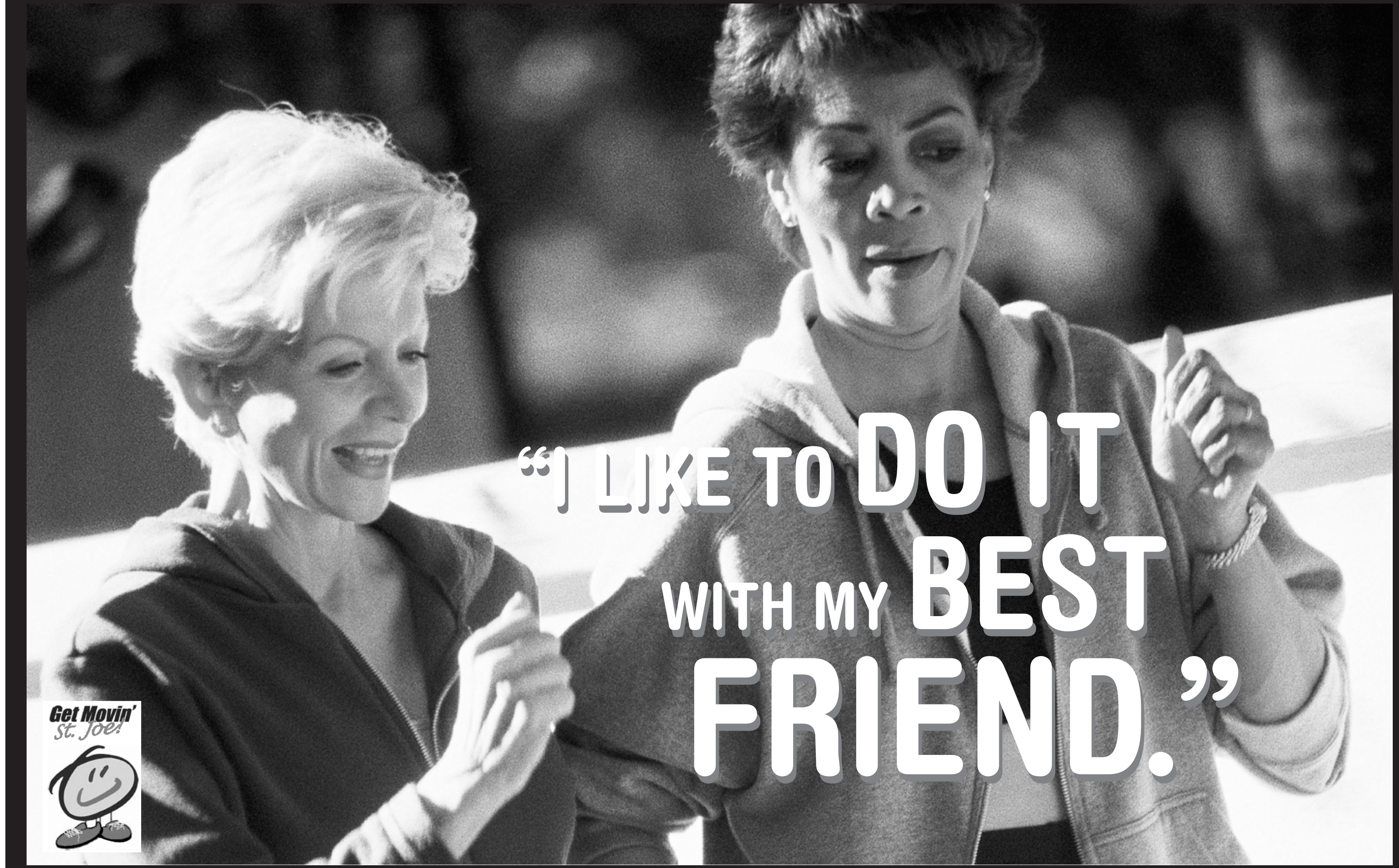

WHO DO
YOU
WALK
WITH?

WALK
MISSOURI

Get Movin' St. Joseph,
Heartland Regional Medical Center,
and
St. Joseph Family YMCA

Invite you to a community walk at the Shelter near the playground at Hyde Park

5:30 PM on Tuesday May 6th & Wednesday May 7th

News from the St. Joseph Family YMCA

WALK REEBOK

Tuesdays & Thursdays starting June 3 at 5:30 - 6:30 p.m.
This collaborative program between YMCA of the USA and REEBOK International Ltd. Includes the latest information on walking technique, new physical screening tests, new stretching and strengthening exercises, mini lectures, participant handouts and walking workouts. YMCA and other locations to be announced by instructor. Don't just walk...workout!

YMCA Members: FREE **Activity Fee (Non-member fee): $48.00**

WATER WALKING

Monday through Friday 9:30 - 10:30 a.m.
Exercise with minimal stress to your joints in our Water Walking class where 30 minutes of water walking is comparable to 1 1/2 hours of walking on land.

Members: FREE **Activity Fee (Non-member fee): $48.00**
